# TAGAP restrains myeloid and T cell activation in inflammatory bowel disease

**DOI:** 10.3389/fimmu.2025.1641365

**Published:** 2025-09-22

**Authors:** Rui-Ci Lin, Zhifei Shao

**Affiliations:** Inflammation Research, Amgen Inc., South San Francisco, CA, United States

**Keywords:** TAGAP, inflammatory bowel disease (IBD), GTPase activating protein, innate immunity, Th17 cells

## Abstract

**Introduction:**

Inflammatory bowel disease (IBD) is characterized by chronic, relapsing inflammation of the gastrointestinal tract. Genetic factors, including variants in T-cell activation Rho GTPase-activating protein (TAGAP), contribute to disease susceptibility and severity.

**Methods:**

A newly identified TAGAP coding variant E147K was examined by GTP hydrolysis assay. To clarify the role of TAGAP in human immune regulation, CRISPR knockout approaches were employed to study functional changes of human myeloid and T cells. Murine studies, including anti-CD3 antibody injection and CD4^+^CD45RB^hi^ T cell adoptive transfer models, were conducted to assess TAGAP-mediated Th17 responses and chronic colitis severity.

**Results:**

IBD-risk-associated TAGAP variant E147K exhibited enhanced GTPase-activating activity. In human innate and adaptive immune cells, TAGAP restrained cell activation, migration, phagocytosis, and proinflammatory cytokine release. TAGAP deficiency led to significantly increased Th17 cell accumulation due to reduced apoptosis in the murine small intestine and exacerbated chronic intestinal inflammation.

**Discussion:**

These findings demonstrated the role of TAGAP in immune responses and homeostasis. The E147K variant underscores TAGAP’s protective function in IBD, and further investigation is warranted to translate these insights into therapeutic strategies for autoimmune diseases.

## 1 Introduction

Inflammatory bowel disease (IBD), including Crohn’s disease (CD) and ulcerative colitis (UC), is characterized by chronic inflammation of the gastrointestinal tract. Although its etiology remains incompletely understood, IBD is widely considered to result from a complex interplay of genetic susceptibility and environmental factors, culminating in aberrant immune responses against the intestinal microbiota (1, 2). IBD GWAS has identified multiple disease-associated single nucleotide polymorphisms (SNPs), among which missense variants allow us to assess the impact of the mutation on protein function. This provides direct evidence for unraveling disease pathogenesis and identifying novel therapeutic targets tailored to individual patients.

T-cell activation RhoGTPase-activating protein (TAGAP), encoded on human chromosome 6 (3), is predominantly expressed in leukocytes and serves as a critical regulator of immune responses. TAGAP converts GTP-bound Rho GTPases to their inactive GDP-bound state. Loss-of-function (LoF) in GTPase activating protein (GAP) prolongs GTPases in the active form, thereby affecting actin polymerization, cell migration, and phagocytosis (4, 5). Conversely, gain-of-function (GoF) mutations in GAP accelerate GTP hydrolysis, thus reducing active Rho GTPase levels. As a member of the Rho GAP superfamily, TAGAP has emerged from genome-wide association studies (GWAS) as a key locus linked not only to IBD but also to other autoimmune disorders, including multiple sclerosis (MS) and rheumatoid arthritis (RA) (3, 6–9). Evidence from animal models, such as DSS-induced colitis (10), experimental autoimmune encephalomyelitis (EAE) (11, 12), and collagen-induced arthritis (CIA) (3), corroborates the involvement of TAGAP in autoimmune pathogenesis.

Functional studies indicate that TAGAP participates in cytoskeletal remodeling, modulating sperm motility and T cell activation (6, 13). While some reports suggest that TAGAP regulates murine Th17 differentiation, results have been inconsistent across different experimental designs (11, 12). Moreover, a comprehensive understanding of TAGAP function in different human immune cells remains limited. Although TAGAP-mediated Dectin-1 signaling has been reported in murine macrophages (12), the lack of in-depth understanding of its role in myeloid cell function and broader immune regulation warrants further exploration.

In this study, we reported a CD-associated coding variant in TAGAP conferring a GoF phenotype by enhancing GTPase activity. We further demonstrated that TAGAP suppresses activation and effector functions in both human myeloid and lymphoid cells and curtails Th17 cell expansion *in vivo*. Moreover, we also observed that TAGAP knockout leads to increased inflammation, particularly driven by enhanced Th17 cell responses. Together, our findings provide further insight into the function of TAGAP and the rationale for TAGAP being a leukocyte-specific therapeutic target for IBD.

## 2 Materials and methods

### 2.1 Ethics approval and consent to participate

All methods performed in this study adhered to the relevant guidelines and regulations. Human specimens were collected in accordance with the specifications of the Amgen Human Tissue Science Center study, under the approval of site-specific Institutional Review Boards or Ethics Boards, with informed consent obtained in compliance with all applicable laws and regulations.

### 2.2 Generation of *Tagap* knockout mice

*Tagap* knockout mice were generated using CRISPR-based extreme genome editing (EGE™) technology by Biocytogen Pharmaceuticals. Two sgRNAs targeting the intron 4 and intron 7 were designed to mediate the deletion of 2.3kb chromosomal region (Exon5, Exon6, and Exon7) at the *Tagap* locus in the mouse genome.

Briefly, two sgRNAs were designed by the CRISPR design tool (http://cctop.cos.uni-heidelberg.de:8043/) and then were screened for on-target activity using a Universal CRISPR Activity Assay (UCA™, Biocytogen Pharmaceuticals). The T7 promoter sequence was added to the sgRNA template by PCR amplification *in vitro*.

The nuclease mRNA and two sgRNAs were co-microinjected into the cytoplasm of one-cell stage fertilized C57BL/6J eggs and then the injected embryos were transferred into oviducts of pseudopregnant females to generate F0 mice. Of 866 zygotes transferred, 131 pups were obtained, of which 6 F0 mice were confirmed as positive founders. F0-positive founders were bred with wild-type (WT) C57BL/6J mice to generate F1 pups. Germline transmission had been achieved from founder line E2S0192-0019. F1 heterozygous mice were confirmed by two rounds of tail genomic PCR and DNA sequencing.

### 2.3 Animals

Six- to eight-week-old male and female WT and TAGAP KO mice on C57BL/6 background and female Rag2^-/-^ mice purchased from Taconic Biosciences were used in this study. All the mice were maintained on a 12-hour light/dark cycle under specific pathogen-free conditions with access to food and water *ad libitum*. Protocols were approved by the Institutional Animal Care and Use Committee of Amgen.

### 2.4 GAP assay

All recombinant TAGAP proteins (WT, K147, and A123) were produced and purchased from WuXi Biologics. The sequence design is attached in **Supplementary Table 1**. GAP activity was determined with the RhoGAP assay kit (Cytoskeleton, Inc.; #BK105). Briefly, GTPase activity was measured by detecting inorganic phosphate (Pi) released from GTP hydrolysis with or without GAP molecules in the reaction. The reactions were initiated with the addition of 200 µM GTP to a RhoGTPase in the presence or absence of TAGAP at 37 °C for 5 min, followed by adding CytoPhos reagent. The absorbance at 650 nM indicated the concentration of released phosphate and was recorded at 2-min intervals for 1 hr to monitor reaction kinetics.

### 2.5 Western blotting

Cells were lysed in RIPA buffer (ThermoFisher; #89900) in the presence of a protease inhibitor cocktail (ThermoFisher; #78429). Protein concentrations in cell lysates were quantified using the BCA assay (ThermoFisher; #23225). Thirty μg of total protein lysate were used for Western blotting. Western blotting was performed using 4-12% Bis-Tris gels, followed by transferred onto NC membranes. After blocking with 5% skim milk powder, the membranes were incubated overnight at 4°C with primary antibodies at 1:1000 dilution and secondary antibodies were used at 1:10000 dilution at room temperature for 2 h. All primary and secondary antibodies are listed in **Supplementary Table 2**. Protein samples were visualized using a ChemiDoc XRS+ imaging system (BioRad).

### 2.6 Generation of TAGAP KO primary cell and cell line

Three single guide RNAs (sgRNAs) targeting the TAGAP gene as well as two non-targeting (NT) control sgRNAs were predesigned and purchased from IDT. The sequences of the sgRNAs are shown in **Supplementary Table 3**. Transfection was performed using the Neon Transfection System (ThermoFisher; #MPK10025 and MPK1025) according to the manufacturer’s guide and protocols. Briefly, human primary monocytes/pan T cells/HL-60/THP-1/Jurkat cells were used in individual reactions mixed with sgRNA-nuclease ribonucleoprotein complexes. Knockout efficiency was confirmed by Western blotting. KO pool was used to minimize clonal bias in each experiment.

### 2.7 Migration assay

HL-60 cells were differentiated in 1.3% DMSO for 6 days as described before (14). Two hundred and fifty thousand differentiated cells in RPMI containing 0.5% BSA were then seeded in inserts of transwell plates (Corning; #3421) with the bottom wells loading different concentrations of fMLP (Sigma; #F3506) or LTB4 (TOCRIS; #F3506) depending on experimental settings. After the incubation at 37°C for 45 min, migrated cells (cells in the bottom wells) were quantified with CellTiter-Glo (Promega; #G7570) per manufacturer’s protocols.

### 2.8 Phagocytosis assay

The phagocytic activity of HL-60 cells was measured by the uptake of opsonized red fluorescent pHrodo Escherichia coli bioparticles (Invitrogen; #P35361) as described by Jun et al. with modification (15). Briefly, 5 × 10^4^ DMSO-induced differentiated HL-60 cells were mixed with pHrodo *E. coli* bioparticles at a 1-to-30 ratio in HBSS containing 10 mM HEPES and 5% FBS. The mixture was incubated at 37°C and the kinetic uptake activity was recorded and analyzed using the Incucyte live cell imaging and analysis system (Sartorius).

### 2.9 Human primary monocytes and T cells isolation

Human PBMCs were purified from human whole blood using LeucoSep tubes according to the manufacturer’s recommendations. Briefly, 2X diluted whole blood with PBS was overlaid on the separation medium Ficoll (Cytiva, #17144003) in LeucoSep tubes (Greiner, #227290). Samples were spun at 800g with low acceleration and low brake for 20 min. After centrifugation, the buffy coat was collected for further purification using EasySep Human Monocyte or T Cell Isolation Kits (STEMCELL; #19359 or #17951, respectively).

### 2.10 Human monocyte differentiation and stimulation

Purified human monocytes were transfected as described above to generate NT and TAGAP KO cells. After the electroporation, cells were rested in RPMI containing 10% FBS and 50 ng/ml M-CSF (Gibco; # 300-25) for overnight. Cells were then cultured in fresh M-CSF-supplemented media for 7 days with replenishment of fresh media on day 3 and 5. On day 7, cells were harvested and stimulated with TLR or Dectin-1 ligands for 24 hr. All TLR and Dectin-1 ligands are listed in **Supplementary Table 4**. After the stimulation, cells were harvested for Western blotting and supernatant was collected for MSD cytokine/chemokine analysis.

### 2.11 Jurkat line generation and stimulation

Jurkat and Jurkat-NFAT-GFP cells were transfected as described above to generate NT and TAGAP KO cells. For TCR-dependent stimulation, Jurkat lines in RPMI containing 10% FBS were seeded in aCD3-coated plates (Invitrogen; #16003785; concentration as each experiment indicated) with 1 µg/ml aCD28 (Invitrogen; #16028985) in suspension for 24hr. For TCR-independent stimulation, Jurkat lines in RPMI containing 10% FBS were cultured in the presence of 10 ng/ml PMA (InvivoGen; # tlrl-pma) and 500 ng/ml Ionomycin (InvivoGen; # inh-ion) for 24hr. Cells were then collected to test the activation marker expression and GFP levels via flow cytometry.

### 2.12 Plasmid transfection of TAGAP-deficient Jurkat cells

TAGAP KO Jurkat cells were transfected with constructs encoding WT TAGAP E147, the K147 variant, or the A123 variant, each tagged with a Myc-DDK tag at the C-terminus and cloned into the CD811A backbone vector (System Biosciences, #CD811A-1). An empty vector was used as a control. Transfections were performed using the SE Cell Line 4D-Nucleofector X Kit (Lonza, #V4XC-1032) following the manufacturer’s protocol. Briefly, 1 × 10^6^ cells were resuspended in 20 μl of Nucleofector Solution mixed with **Supplementary Data Sheet 1** and 1 μg of plasmid DNA per reaction. Electroporation was carried out using the 4D-Nucleofector System (Lonza), and cells were immediately transferred to pre-warmed RPMI 1640 supplemented with 10% FBS. Transfected cells were rested for 48 hours prior to stimulation and downstream assays as described above.

### 2.13 Human primary T cell stimulation

Purified human pan T cells were transfected as described above to generate NT and TAGAP KO cells. Cells in RPMI containing 10% FBS and β-ME were seeded in 1 ug/ml aCD3-coated plates (Invitrogen; #16003785) with 1 µg/ml aCD28 (Invitrogen; #16028985) in suspension. Cells and supernatant were collected at 24-hr- and 72-hr-post-stimulation to test the activation marker expression via flow cytometry and cytokine levels via ELISA.

### 2.14 Intraperitoneal anti-CD3 injection animal model

Th17 cell composition in small intestines (SI) from anti-CD3-challenged mice was determined by flow cytometry as described by Esplugues et al. previously (16). In short, WT and TAGAP KO mice were injected with anti-CD3 (15 µg; BioXCell; #BE0001-1) intraperitoneally (*i.p.*) three times at an interval of 2 days between injections and sacrificed 4 hr after the final injection. For the controls, isotype control antibodies (15 µg; BioXCell; # BE0091) were injected. The serum and the distal 2 cm of SI for tissue homogenate were collected for cytokine/chemokine measurement. The IEL and LPL from SI were collected with the lamina propria dissociation kit (Miltenyi Biotec; #130-097-410) according to the manufacturer’s manual. The dissociated IEL and LPL cells were loaded onto a Percoll gradient and centrifuged at 2000 rpm for 20 min. The cells between 32.5% and 80% Percoll were collected and used as purified IEL and LPL. Both purified IEL and LPL were stimulated with the cell stimulation cocktail (Invitrogen; #00-4970-93) in the presence of the protein transport inhibitor (Invitrogen; #00-4980-03) at 37°C for 4 hr prior to the intracellular staining for flow cytometry.

### 2.15 CD4^+^CD45RB^hi^-mediated chronic colitis model

CD4^+^ T cells from spleens of WT or TAGAP KO mice were collected and purified by EasySep mouse CD4^+^ T cell isolation kits (STEMCELL; #19852). After the enrichment, total CD4^+^ T cells were FACS sorted and 5×10^5^ CD45RB^hi^ T cells were adoptively transferred (*i.p.*) into female Rag2^-/-^ recipient mice. Body weight and survival were monitored daily for 60 consecutive days. Harvested SI were processed for flow cytometry analysis as described above.

### 2.16 Flow cytometry and Annexin V staining

Single-cell suspensions were blocked, Live/Dead stained, surface stained, washed, fixed, and permeabilized then intracellularly stained as previously described (17). All flow antibodies used in the study are listed in **Supplementary Table 2**. Annexin V staining was performed per the manufacturer’s recommendation (Biolegend; #640906). Flow cytometry was performed on BD Symphony A3 Cytometer.

### 2.17 MSD and ELISA

V-PLEX Proinflammatory Panel 1 Human Kit (MSD; #K15049D-1), Human IL-2 ELISA kit (Invitrogen; BMS2212), and Humun IFN-γ quantikine ELISA kit (R&D Systems; #DIF50C) were used to measure the cytokine/chemokine levels from the supernatant of cultured human macrophages and T cells. U-PLEX Proinflam Combo 1 mouse kit (MSD: #K15713K-1) and mouse CCL20 quantikine ELISA kit (R&D Systems; #MCC200) were used to measure the cytokine/chemokine levels from mouse serum and SI tissue homogenate following the instruction from the manufacture.

### 2.18 Fluorescent confocal microscopy

Differentiated NT and TAGAP KO human macrophages were seeded into poly-D-lysine-coated glass-bottom culture slides (Corning, #354632) and stimulated with 100 ng/ml LPS for 24 hr. Following stimulation, cells were fixed with 4% paraformaldehyde (PFA) and permeabilized with 0.1% Triton X-100. After blocking with 1% bovine serum albumin (BSA) for 1 hr at room temperature, cells were stained with a cocktail of Phalloidin-FITC (Invitrogen, #F432) and DAPI (Invitrogen, #D1306) for 1 hr. Slides were washed three times with PBS, air-dried, and mounted using anti-fade mounting medium. Fluorescent images were acquired using a Leica SP8 confocal microscope.

### 2.19 Exploration of gene expression in public RNAseq datasets

Expression data from the cell lines were extracted from QIAGEN OmicSoft Cell Line release CellLine_B38_GC33, and expression data from primary human immune cells were extracted from Body Map release Blueprint_B37. Data were analyzed and visualized using QIAGEN OmicSoft Studio software, version 12.4 (QIAGEN, Redwood City, CA).

### 2.20 Statistical analysis

The data are shown as mean ± S.D. from at least three independent experiments including technical and biological replicates. The p values noted in the corresponding figure legends were calculated using Student’s t-test (among two groups) or one-way ANOVA (among multiple groups) in Prism (GraphPad Software 8.0, La Jolla, CA).

## 3 Results

### 3.1 TAGAP E147K leads to a protein gain-of-function

The involvement of TAGAP in IBD has been demonstrated in both animal and human studies (8–10, 18). Common non-coding variants in TAGAP locus have been identified in GWAS to be associated with the risk of Crohn’s disease (CD) (19), but it is still unclear whether the association is driven by gain or loss of function of TAGAP. A recent exosome sequencing study identified a rare missense variant rs41267765 C/T in TAGAP associated with CD risk (20). The protective allele (T) introduces a Glu (E) to Lys (K) substitution at residue 147, yielding an odds ratio of 0.786 and suggesting that TAGAP E147K is protective against CD. Given the evidence that TAGAP deletion exacerbates DSS-induced colitis severity, we hypothesized that the E147K mutation reduces IBD risk by altering TAGAP function.

**Figure 1:**
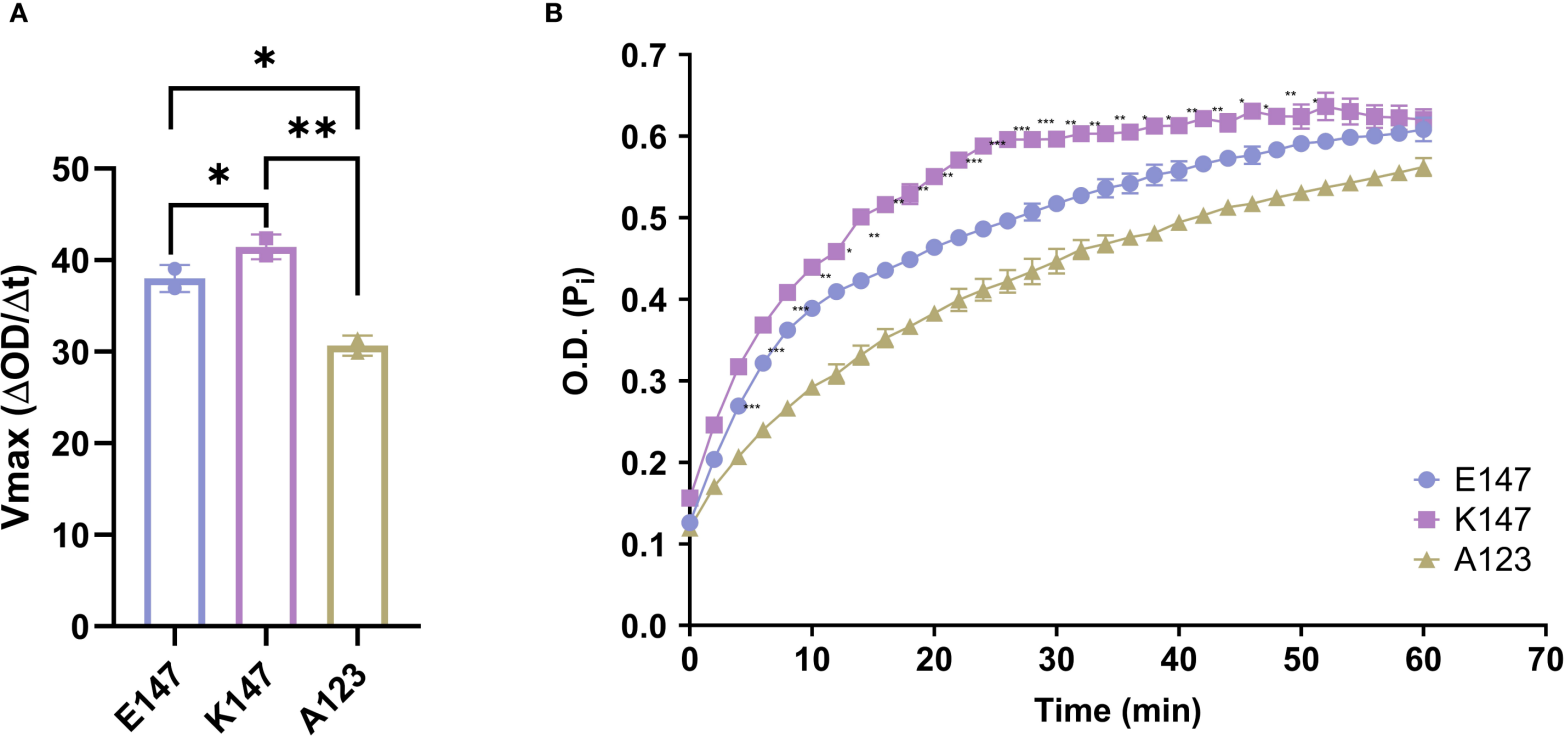
E147K substitution promotes TAGAP activity. **(A)** Vmax of E147, K147, and A123 TAGAP variants facilitated RhoA-dependent GTP hydrolysis reactions (n = 2; **P < 0.01, *P < 0.05 using one-way ANOVA test followed by the *post-hoc* Sidak multiple comparisons test). **(B)** Time course of inorganic phosphate production from TAGAP variants facilitated GTP hydrolysis reactions (n = 2; ***P < 0.001, **P < 0.01, *P < 0.05 using one-way ANOVA test followed by the *post-hoc* Sidak multiple comparisons test between E147 vs. K147). Each dot represents an individual reaction. All data are presented as mean ± SD.

To investigate this hypothesis, we acquired three human recombinant TAGAP proteins: wild-type (WT; E147), K147, and a negative-control A123. TAGAP A123 has decreased TAGAP activity as it harbors an Arg-to-Ala mutation at residue 123, a critical site for Rho protein interaction. We then assessed TAGAP-facilitated GTP hydrolysis by measuring the release of inorganic phosphate (Pi) over time. Compared with the WT protein, the K147 mutant facilitated GTP hydrolysis with an approximately 14% increase in Vmax (**Figures 1A, B**). Conversely, the A123 mutation significantly impaired TAGAP function, reducing Vmax by ~20% (**Figures 1A, B**). These findings indicated that the E147K mutation enhances TAGAP-dependent GTP hydrolysis activity, conferring a gain-of-function phenotype.

### 3.2 TAGAP is expressed in monocytes and suppresses myeloid cell migration, phagocytosis, and cytokine secretion

**Figure 2:**
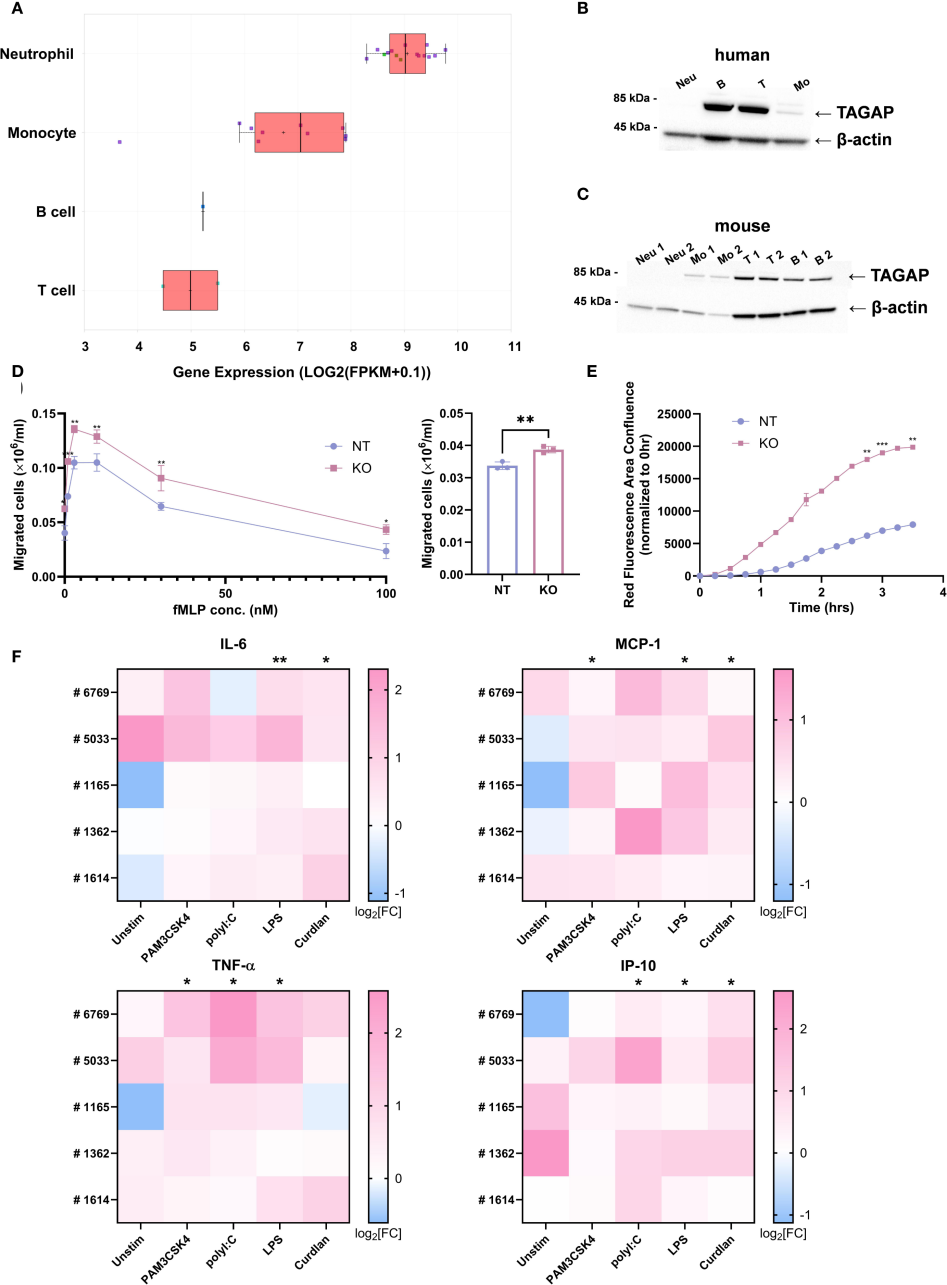
TAGAP is expressed in myeloid cells and plays a suppressive role in myeloid cell migration, phagocytosis, and cytokine secretion. **(A)** Box plot comparing *TAGAP* expression in different human primary immune cells in QIAGEN Omicsoft Body Map release Blueprint_B37. **(B)** Western blotting of TAGAP from different human blood immune cells. **(C)** Western blotting of TAGAP from murine bone-marrow-derived neutrophils and monocytes as well as splenic T cells and B cells. **(D)** The migrated non-targeting (NT) control and TAGAP KO HL-60 cells in response to fMLP gradient (left) and 10 nM LTB4 (right) after 60-min incubation in transwell systems (n = 3; **P < 0.01, *P < 0.05 using Student’s t-test). **(E)** Kinetics of NT control and TAGAP KO HL-60 cell phagocytosis in the cell and pHrodo Red *E*. *coli* BioParticle coincubation assay. The area of red fluorescence emitting *E*. *coli* BioParticles was captured and analyzed by Incucyte at different time points (n = 2; ***P < 0.001, **P < 0.01 using Student’s t-test). **(F)** Assessment of IL-6, MCP-1, TNF-α, and IP-10 content from human monocyte-derived macrophage cultures. Blood monocytes from five individual donors (#6769, #5033, #1165, #1362, and #1614) were purified, electroporated with NT or TAGAP sgRNA, and differentiated, followed by 100 ng/ml PAM3CSK4, 100 ng/ml polyI:C, 100 ng/ml LPS, or 100 µg/ml Curdlan stimulation for 24hr. Secretion of the cytokines was quantified using MSD. For each donor and condition, cytokine levels from TAGAP KO cells were normalized to their corresponding NT counterparts. Data are presented as log2 fold changes (KO/NT). Statistical significance was determined by comparing the normalized KO/NT fold change against a baseline value of 1 (NT/NT) (n = 5; **P < 0.01, *P < 0.05 using Student’s t-test). Each dot represents an individual reaction. All data are presented as mean ± SD. Neu, neutrophils; B, pan B cells; T, pan T cells; Mo, monocytes; NT, non-targeting control; KO, TAGAP knockout.

Analysis of publicly available RNA expression data from QIAGEN Omicsoft Body Map release Blueprint_B37 showed that TAGAP is exclusively expressed in leukocytes, with neutrophils exhibiting the highest RNA transcript levels (**Figure 2A**). However, at the protein level, TAGAP is more abundant in human (**Figure 2B**) and murine lymphocytes (**Figure 2C**) than in myeloid cells, with moderate expression in monocytes and undetectable levels in neutrophils.

To better understand the biology of TAGAP in leukocytes, first we sought to determine TAGAP’s function in myeloid cells. HL-60 cells, a promyeloblast line, were selected due to their relatively high TAGAP expression compared with THP-1 cells (**Supplementary Figure 1A**), a finding confirmed at the protein level via western blot (**Supplementary Figure 1B**). To explore whether TAGAP regulates myeloid cell functions, CRISPR-mediated knockout (KO) of TAGAP was performed in HL-60 cells (**Supplementary Figure 1B**), followed by transwell assays to assess cell migration. Compared to controls, TAGAP KO cells displayed significantly increased migration in response to both fMLP and LTB4 (**Figure 2D**). Phagocytic activity was also enhanced in KO cells, as indicated by 2- to 4-fold higher fluorescence from pHrodo-labeled *E. coli* BioParticles over time (**Figure 2E**). These observations suggested that TAGAP constrains both migration and phagocytosis in this myeloid cell model.

Human PBMC-derived macrophages were subsequently used to further investigate TAGAP’s role in primary myeloid cells (**Supplementary Figure 2A**). Following knockout and stimulation with PAM3CSK4 (TLR1/2 ligand), poly(I:C) (TLR3 ligand), LPS (TLR4 ligand), or Curdlan (Dectin1 ligand), KO macrophages released higher levels of IL-6, MCP-1, TNF-α, and IP-10 than non-targeting (NT) controls, especially upon LPS stimulation (**Figure 2F**). Although macrophages also produced IL-1β and IL-23 in response to curdlan stimulation, their secretion of these cytokines was lower in KO than in NT cells, consistent with the prior findings (**Supplementary Figure 2B**) (12).

To further examine the involvement of TAGAP in cytoskeletal remodeling, we performed fluorescent confocal imaging of TAGAP KO and NT control macrophages stained with DAPI (nuclei) and Phalloidin-FITC (F-actin). Under the unstimulated condition, no major differences in morphology were observed. Following LPS stimulation, TAGAP KO macrophages exhibited a heterogeneous morphology characterized by a mixture of spindle-shape and dendrite-like cells, whereas NT macrophages primarily displayed a rounded morphology with radiating dendritic extensions (**Supplementary Figure 3**). These changes suggested that TAGAP modulates cytoskeletal architecture in macrophages, consistent with previous findings that TAGAP regulates thymocyte adhesion and cytoskeletal structure through integrin activation (5). Collectively, these results indicated that TAGAP acts as a negative regulator of human myeloid cell functions and possibly contributes to cytoskeleton dynamics during activation.

### 3.3 TAGAP suppresses human T cell activation

**Figure 3:**
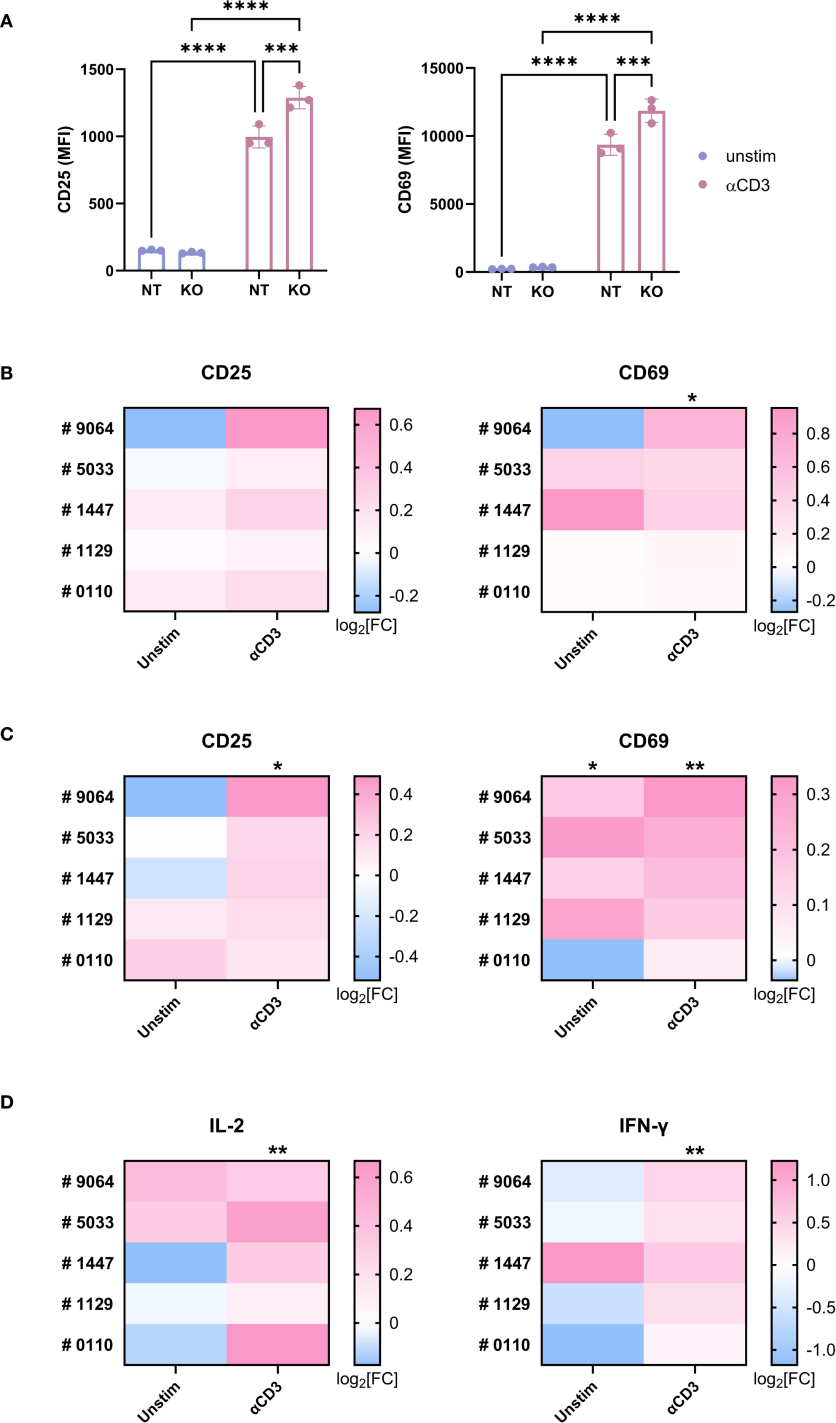
TAGAP is suppressive for human T cell activation. **(A)** The expression of CD25 and CD69 on non-targeting (control) or TAGAP KO Jurkat cells stimulated with 1 µg/ml anti-CD3 antibodies for 24hr (n = 3; ****P < 0.0001, ***P < 0.001 using two-way ANOVA test followed by the *post-hoc* Fisher’s LSD multiple comparisons test). **(B, C)** The expression of CD25 and CD69 on human primary CD4^+^ **(B)** and CD8^+^ T cells **(C)**. Blood T cells from five individual donors (#9064, #5033, #1447, #1129, and #0110) were purified and electroporated with non-targeting (NT) or TAGAP sgRNA, followed by 1 µg/ml anti-CD3 antibody stimulation for 24hr. Expression of the markers was measured (gMFI) by flow cytometry. For each donor and condition, gMFI levels of TAGAP KO cells were normalized to their corresponding NT counterparts. Statistical significance was determined by comparing the KO/NT fold change against a baseline value of 1 (NT/NT). Data are presented as log2 fold changes (KO/NT). (n = 5; **P < 0.01, *P < 0.05 using Student’s t-test). **(D)** The release of IL-2 and IFN-γ from human T cell cultures 24-hr and 72-hr post stimulation, respectively. Blood T cells from two individual donors (#9064, #5033, #1447, #1129, and #0110) were purified and electroporated with non-targeting (NT) or TAGAP sgRNA, followed by 1 µg/ml anti-CD3 antibody challenge. Secretion of the cytokines was quantified using MSD. For each donor and condition, cytokine levels from TAGAP KO cells were normalized to their corresponding NT counterparts. Statistical significance was determined by comparing the KO/NT fold change against a baseline value of 1 (NT/NT). Data are presented as log2 fold changes (KO/NT). (n = 5; **P < 0.01 using Student’s t-test). Each dot represents an individual reaction. All data are presented as mean ± SD. NT, non-targeting control; KO, TAGAP knockout.

Next, we examined the role of TAGAP in T cell responses. TAGAP knockout in Jurkat cells led to increased TCR-mediated activation, indicated by ~1.2-fold higher CD25 and CD69 expression (**Figure 3A**). This finding was corroborated in a Jurkat-NFAT-GFP reporter line, where KO cells exhibited stronger GFP signals in response to both anti-CD3 (~1.3-fold) and PMA/Ionomycin (~1.4-fold) stimulation (**Supplementary Figures 1B**, **4A**).

A similar trend was also observed in primary human T cells (**Supplementary Figure 4B**). Knocking out TAGAP significantly increased CD25 and CD69 levels in both CD4^+^ (**Figure 3B**) and CD8^+^ (**Figure 3C**) T cells upon anti-CD3 stimulation. In addition, KO pan T cells secreted higher amounts of IL-2 and IFN-γ at Days 1 and 3 post anti-CD3 stimulation (**Figure 3D**).

To further investigate whether the biochemical phenotype of TAGAP variants correlates with functional outcomes, we transfected TAGAP-deficient Jurkat cells with constructs encoding TAGAP WT E147, K147, or negative control A123. While overexpression of TAGAP E147 and A123 had a marginal impact on cell activation, overexpression of the K147 variant significantly suppressed CD25 and CD69 expression in Jurkat cells following TCR activation (**Supplementary Figure 4C**). This finding corroborated our biochemical assay data, indicating that TAGAP K147 is a GoF variant with enhanced suppressive function in a cellular context. These data together demonstrated a suppressive role of TAGAP in human T cell activation.

### 3.4 TAGAP regulates Th17 cell expansion in an anti-CD3-challenged murine model

TAGAP has been implicated in T cell differentiation, especially in the generation of Th17 cells; however, conflicting evidence has emerged. Some studies suggest that TAGAP is critical for intrinsic Th17 development (11), whereas others attribute its influence to innate immune signaling in myeloid cells (10, 12). Another line of research indicates that TAGAP interference mitigates CIA severity in rats by downregulating the Th17 response (7).

**Figure 4:**
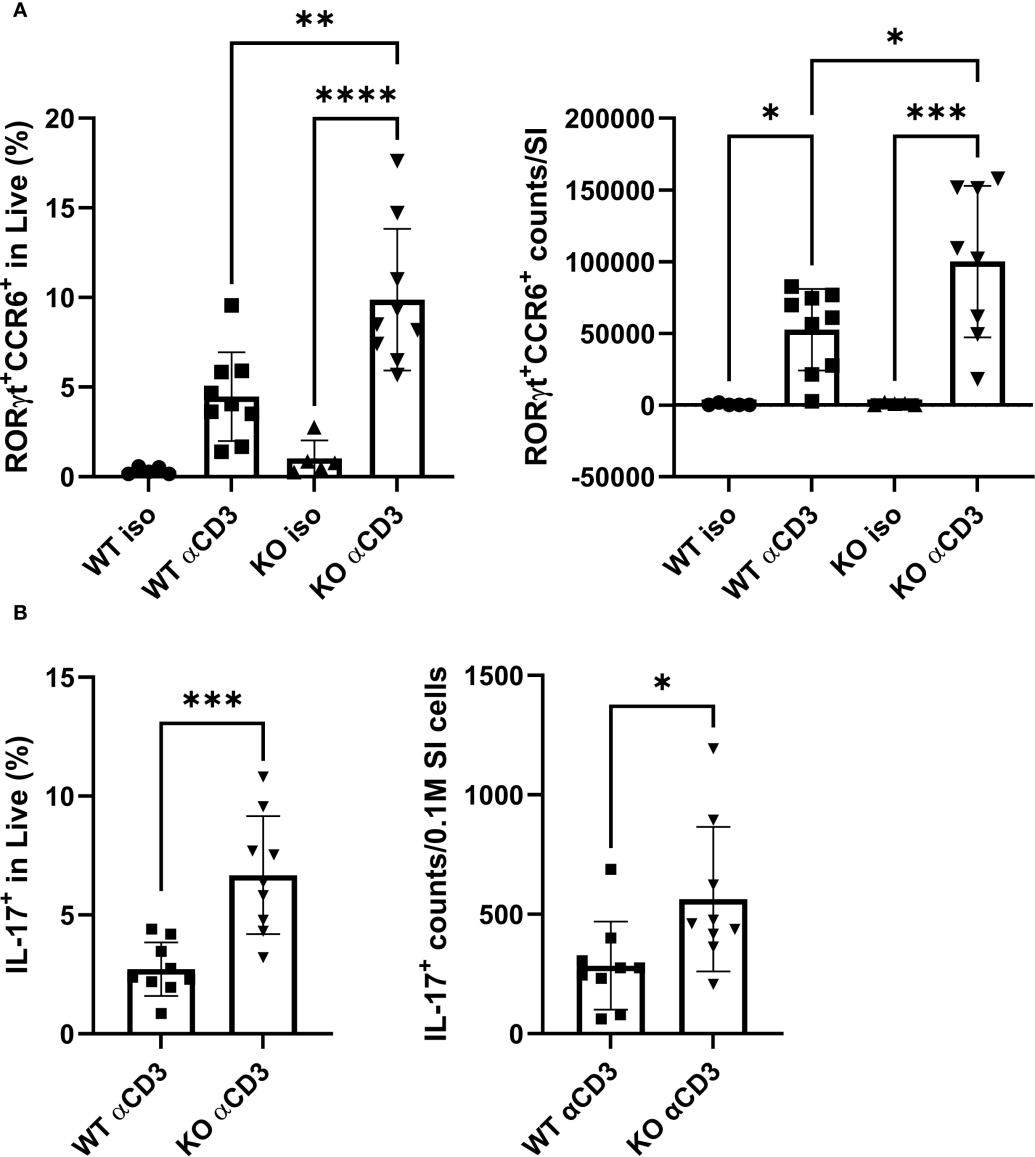
TAGAP regulates Th17 cell expansion in SI LP in response to *in vivo* TCR stimulation. **(A)** The percentage (left) and counts (right) of RORγt^+^CCR6^+^ cells in SI LP from WT or TAGAP KO mice subjected to isotype control antibodies or anti-CD3 antibodies (n = 5 or 9; **P < 0.01, *P < 0.05 using one-way ANOVA test followed by the *post-hoc* Sidak multiple comparisons test). **(B)** The percentage (left) and counts (right) of IL-17A^+^ cells in SI LP. SI LPL from WT or TAGAP KO mice subjected to isotype control antibodies or anti-CD3 antibodies were *ex vivo* stimulated with a cell stimulation cocktail for 4 hr (n = 9; ***P < 0.001 using Student’s t-test). All n-numbers represent data derived from separate mice with data plotted as mean ± SD. WT, wild-type; KO, TAGAP-deficient; iso, isotype control antibodies. ****p<0.0001.

To reconcile these discrepancies and ensure biological relevance, an *in vivo* anti-CD3 (aCD3) *i.p.* injection model was used. In this system, aCD3 depletes circulating T cells but expands the Th17 population in the small intestine (SI) when T cells repopulate. As expected, mice receiving aCD3 possessed more RORγt^+^CCR6^+^ cells in both SI lamina propria (LP) and intraepithelial layer than animals injected with an isotype control antibody (**Figure 4A**; **Supplementary Figure 5A**; the corresponding gating strategies shown in **Supplementary Figure 6**). Notably, KO mice exhibited nearly doubled proportion and absolute count of RORγt^+^CCR6^+^ cells in the SI LP compared with their WT counterparts under aCD3 treatment (**Figure 4A**). This difference was confirmed by stimulating total SI lamina propria lymphocytes (LPL) *ex vivo*, revealing ~2-fold higher proportions and absolute numbers of IL-17^+^ cells in KO mice subjected to aCD3 (**Figure 4B**). These results suggested that TAGAP constrains aCD3-mediated Th17 cell expansion *in vivo*.

To identify the underlying mechanism, we measured CCL20 levels in the SI, as CCR6/CCL20 signaling is one of the key drivers of Th17 recruitment (21, 22). Although tissue CCL20 level was elevated by the aCD3 challenge, there were no significant genotype-specific differences (**Supplementary Figure 5B**). These findings indicated that chemokine-dependent cell migration and development may contribute to the overall Th17 response in the SI upon TCR stimulation, but do not fully explain the heightened Th17 cell increase observed in KO mice. Therefore, the possibility of genotype-related differences in T cell apoptosis was explored next. Further analyses of SI LP revealed that the percentage of Annexin V^+^/PI^-^ cells among CD4^+^ cells was ~50% lower in KO mice than in WT controls (**Supplementary Figure 5C**). These findings implied that TAGAP may restrict Th17 cell populations in part by modulating T cell apoptosis within the SI.

### 3.5 TAGAP controls Th17 cell populations in a CD4^+^CD45RB^hi^-mediated chronic colitis model

**Figure 5:**
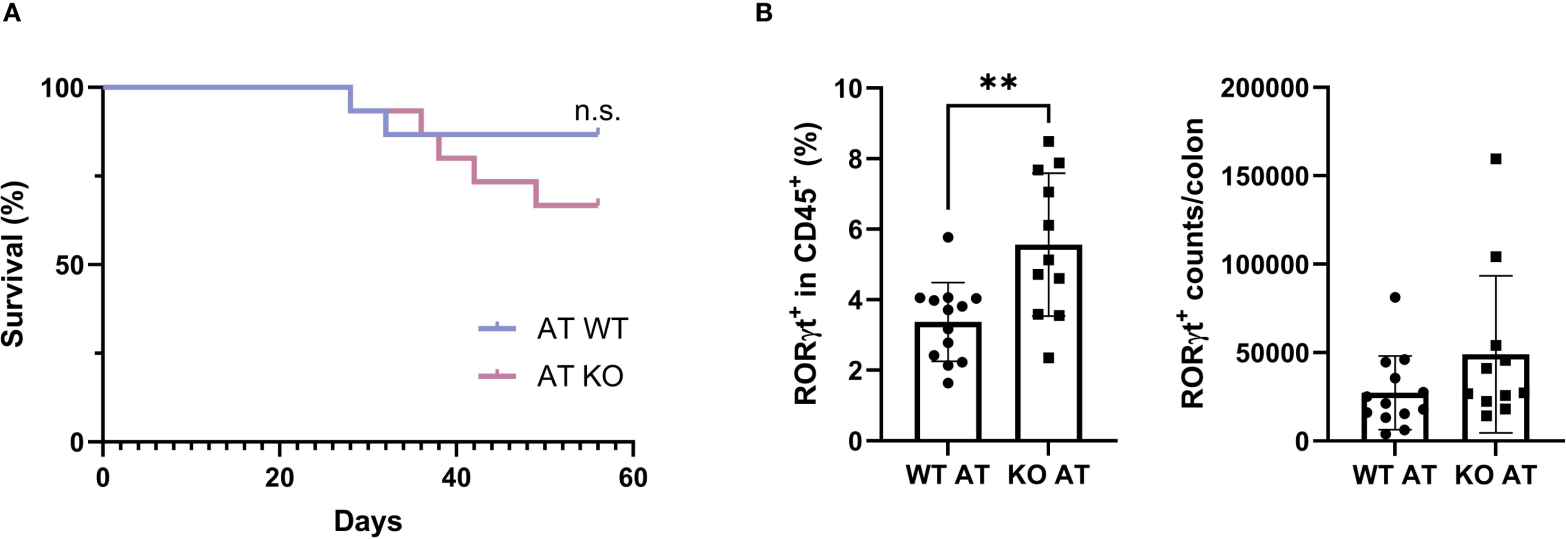
TAGAP controls Th17 cell colonization in the colon of mice with chronic colitis. **(A)** The Kaplan–Meier survival plot of Rag2^-/-^ mice transfused with WT or TAGAP KO CD4^+^CD45RB^hi^ T cells (n = 11 or 13; P value calculated using Log-rank test). **(B)** The percentage (left) and counts (right) of RORγt^+^ cells in colon IEL from Rag2^-/-^ mice transfused with WT or TAGAP KO CD4^+^CD45RB^hi^ T cells (n = 11 or 13; **P < 0.01 using Student’s t-test). All n-numbers represent data derived from separate mice with data plotted as mean ± SD. WT AT, adoptively transferred with wild-type CD4^+^CD45RB^hi^ T cells; KO AT, adoptively transferred with TAGAP-deficient CD4^+^CD45RB^hi^ T cells.

While TAGAP deficiency is known to exacerbate acute colitis (10), its role in chronic colitis remains unclear. To address this gap, a chronic colitis model with CD4^+^CD45RB^hi^ T cell transfer was established in Rag2^-/-^ recipient mice. Recipients were infused with either WT or TAGAP KO donor T cells and monitored over 55 days. Body weight changes were not significantly different between groups (**Supplementary Figure 7**), yet the survival rate was reduced (67%) in mice receiving KO cells compared with those receiving WT cells (87%) (**Figure 5A**), aligning with prior findings in acute colitis models.

Th17 cell frequencies were also examined in the colon given the crucial role of Th17 cells in chronic colitis (corresponding gating strategies shown in **Supplementary Figure 8**). While little difference was noted in LP Th17 cells (**Supplementary Figure 9A**), we focused on intestinal intraepithelial lymphocytes (IEL), which serve as a frontline defense against luminal microbes and help maintain mucosal homeostasis by rapidly responding to epithelial stress and infections (23, 24). Moreover, dysregulation of IEL populations has been implicated in various models of colitis and may potentiate local inflammation (25, 26). Notably, the proportion of RORγt^+^ cells in colon IEL from KO cell recipients was ~2-fold higher than in those receiving WT cells (**Figure 5B**). Correspondingly, the absolute number of RORγt^+^ in colon IEL (**Figure 5B**), as well as IL-17A levels in serum (**Supplementary Figure 9B**) and colonic tissue (**Supplementary Figure 9C**), were elevated by ~1.5- to 2-fold in KO cell recipients. Collectively, these results indicated that TAGAP is essential for limiting colitis severity, potentially by restraining Th17 cell populations in chronic inflammatory settings.

## 4 Discussion

TAGAP has been implicated in IBD pathogenesis (8, 9, 18, 27), yet its underlying mechanisms remain incompletely understood. Most studies have focused on murine T cells, particularly in regulating Th17 responses (3, 7, 10–12), but the function of TAGAP in human immune cells has not been fully characterized. In this study, we found that a recently identified TAGAP polymorphism linked to reduced CD susceptibility confers a GoF phenotype. In addition, we showed that TAGAP suppresses human monocyte and T cell activation and restricts Th17 cell expansion, as evidenced by significantly increased Th17 populations in the small intestine in TAGAP KO mice treated with aCD3. Furthermore, we demonstrated that mice transfused with TAGAP KO CD4^+^CD45RB^hi^ T cells experience more severe colitis than those receiving WT CD4^+^CD45RB^hi^ T cells, aligning with our findings that TAGAP functions as a negative regulator of myeloid- and T-cell-mediated proinflammatory responses.

The current landscape of IBD research has increasingly focused on genetic variants associated with disease susceptibility and severity (1, 20, 28). TAGAP variants, such as the intronic polymorphism rs212388 leading to decreased TAGAP expression in PBMCs, have been linked to IBD risk (8). Although coding variants occur less frequently than noncoding ones, they are often more strongly linked to disease, enabling clearer insights into pathogenic mechanisms (29, 30). Sazonovs et al. recently identified a rare missense variant E147K in TAGAP associated with reduced CD risk (20). Our data confirmed that the E147K mutation enhances TAGAP-facilitated GTP hydrolysis relative to the WT variant, suggesting that elevated TAGAP function exerts a protective effect in CD. These findings aligned with murine data showing that TAGAP deficiency exacerbates colitis severity, thus underscoring the relevance of TAGAP activity in IBD development.

TCR stimulation induces TAGAP expression (5, 6). Beyond T cells, it has been shown that TAGAP undergoes phosphorylation upon the activation of Dectin-1-mediated signaling pathway in macrophages (12). In this study, we confirmed the involvement of TAGAP in both innate and adaptive immune cells, showing that TAGAP suppresses human myeloid and T cell activation by restricting phagocytosis, migration, and proinflammatory cytokine release. A separate study on lung adenocarcinoma (LUAD) corroborates the involvement of TAGAP in human CD4^+^ T cell-mediated cytotoxicity (31), revealing a unique role of TAGAP in T cell-mediated anti-tumor immunity. These findings underscore the need for further research focused on TAGAP to clarify its potential translational relevance.

We extended our investigations to explore the influence of TAGAP on Th17 cell response, which is pivotal in the pathogenesis of autoimmune diseases (32, 33). While previous studies have reported that TAGAP interference reduces Th17-related inflammation in collagen-induced arthritis CIA (7) and that TAGAP deficiency diminishes EAE severity by modulating Th17 responses (11, 12), our findings revealed a more pronounced Th17 induction in the SI of TAGAP KO mice. One of possible explanation for this discrepancy could be owing to fundamental differences in experimental settings: prior studies employed antigen-driven disease models, whereas anti-CD3 injection causes activation-induced cell death followed by the selective repopulation and expansion of CD4 T cells in a heavily Th17-skewed milieu in small intestines (16, 34, 35). Enhanced TCR signaling drove Th17 expansion and infiltration more in the TAGAP KO mice than in the WT mice. Additionally, anti-CD3-elicited Th17 accumulation relies on CCR6-dependent gut homing and occurs independently of overt pathology (36). In our model, aligning with previous reports that highlighted the CCR6/CCL20 axis in aCD3-driven Th17 cell expansion (16), we detected elevated CCL20 in aCD3-treated mice but no genotype-specific differences in its levels, suggesting that CCR6/CCL20 is not the principal driver of enhanced Th17 expansion in TAGAP KO mice. These results prompted us to investigate apoptosis, a key regulator of tissue homeostasis and Th17 cell differentiation (37–39). Notably, aCD3-treated KO mice exhibited significantly fewer apoptotic cells in the small intestine, implying that reduced cell death may contribute to Th17 cell accumulation in the absence of TAGAP. While further studies are needed to fully dissect the molecular pathways involved, our findings suggested that TAGAP plays a pivotal role in limiting Th17 cell expansion upon TCR activation *in vivo*.

While TAGAP deficiency has been shown to exacerbate DSS-induced acute colitis due to significantly increased abundance of IL-17A- and IFN-γ-producing colitogenic CD4^+^ T cells in the gut (10), its role in chronic colitis was unclear. In this study, a CD4^+^CD45RB^hi^ T cell transfer model in Rag2^-/-^ mice revealed that TAGAP deficiency augments chronic colitis severity. Elevated Th17 cell frequencies in the colons of TAGAP KO cell recipients further supported that TAGAP functions as a suppressor of Th17 cell expansion in chronic intestinal inflammation. Additional evidence suggested that factors extrinsic to T cells such as microbiota composition shifts in TAGAP KO mice contribute to Th17 cell expansion (10, 40, 41), aligning with our findings that TAGAP-mediated regulation of the Th17 population can ultimately influence the onset and progression of intestinal inflammation.

One limitation of this study arises from the inherent differences between murine and human TAGAP. In mice, the corresponding residue to human E147 is lysine, underscoring a cautious interpretation when extrapolating our findings to human IBD. A more effective *in vivo* model would be to generate a humanized mouse line by replacing the murine TAGAP gene with the human gene or to CRISPR KI the E147 allele in murine TAGAP. These systems would enhance translational fidelity for modeling human-specific genetic variants in autoimmune diseases. Furthermore, TAGAP is also expressed in B cells, yet its role in this lineage has not been explored to our knowledge, warranting future investigation. Another open question is whether the Th17 cells expanded under TAGAP-deficient conditions are pathogenic and contribute to autoimmunity. Th17 cells are known to both drive intestinal inflammation and support mucosal barrier integrity. Future studies should leverage co-staining for Th17-associated cytokines, such as IL-17A, IL-10, and IFN-γ, alongside transcription factors, such as T-bet or RORγt, to enable comprehensive phenotypic and functional characterization of Th17 subsets. This approach will facilitate the determination of whether TAGAP specifically restricts pathogenic Th17 programming. Together, integrating humanized *in vivo* models with detailed phenotypic profiling of immune cell subsets will clarify the broader role of TAGAP in immune regulation.

Our findings indicated that enhancing TAGAP activity represents a promising therapeutic strategy for CD. Although no small molecule TAGAP activator has been identified to date, potential strategies include molecular-glue-like compounds that promote proximity between TAGAP and its target Rho GTPases as well as compounds that stabilize the TAGAP-RhoGTPase complex to enhance TAGAP-mediated GTP hydrolysis (42), thereby reducing pro-inflammatory signaling cascades. The TAGAP E147K CD-protective variant has not been associated with any reported pathogenic phenotype. This supports the concept that enhanced TAGAP activity is both beneficial and well-tolerated in humans, reinforcing the feasibility and safety of pharmacologic approaches designed to boost TAGAP function.

Together, our findings shed light on the role of TAGAP as a negative regulator in IBD and immune responses. Further investigations are needed to elucidate the molecular mechanisms of TAGAP in human autoimmune diseases and to evaluate its potential as a therapeutic target.

## Data Availability

The raw data supporting the conclusions of this article will be made available by the authors, without undue reservation.
