# Supplementary material for: TAGAP restrains myeloid and T cell activation in inflammatory bowel disease
Source: Front Immunol. 2025 Sep 22;16:1641365. doi: 10.3389/fimmu.2025.1641365 (PMC12498112; doi:10.3389/fimmu.2025.1641365)
Supplement: Supplementary file 1 [file DataSheet1.docx]

**
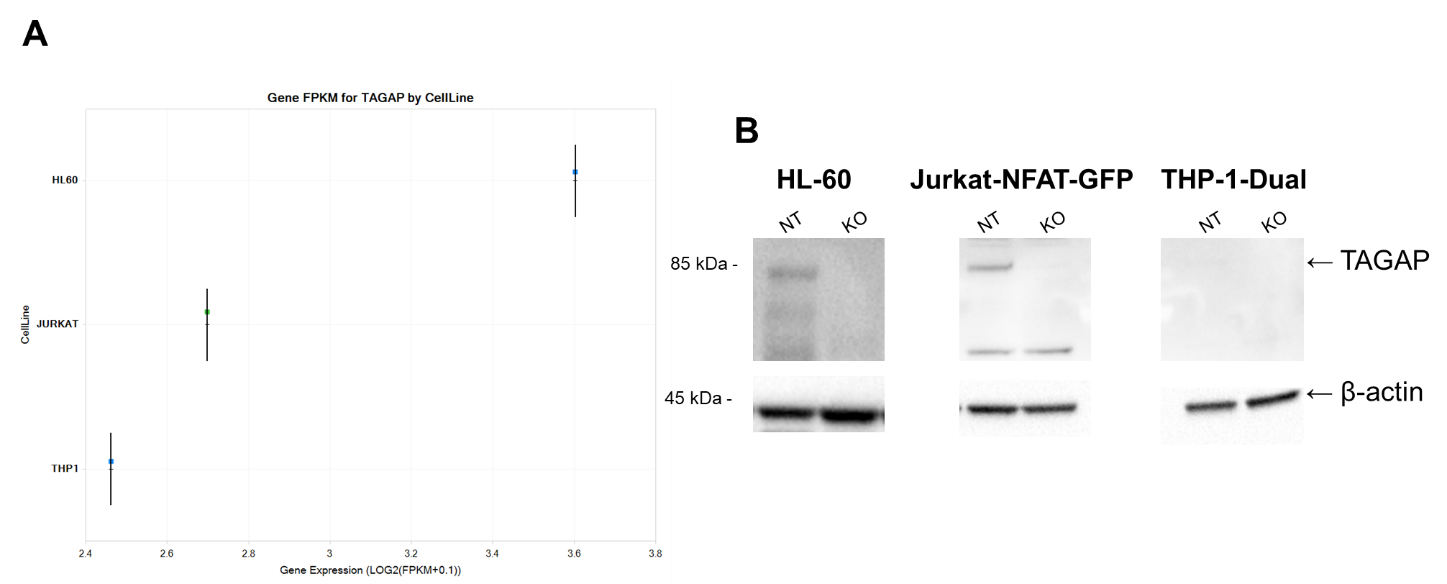
**

**Supplementary Figure 1**

**(A)** Box plot comparing *TAGAP* expression in HL-60, Jurkat, and THP-1 cell lines in QIAGEN Omicsoft Cell Line release CellLine_B38_GC33.

**(B)** Western blotting of TAGAP from HL-60, Jurkat, and THP-1 cell lines following the CRISPR knockout.

NT, non-targeting control; KO, TAGAP knockout.

**
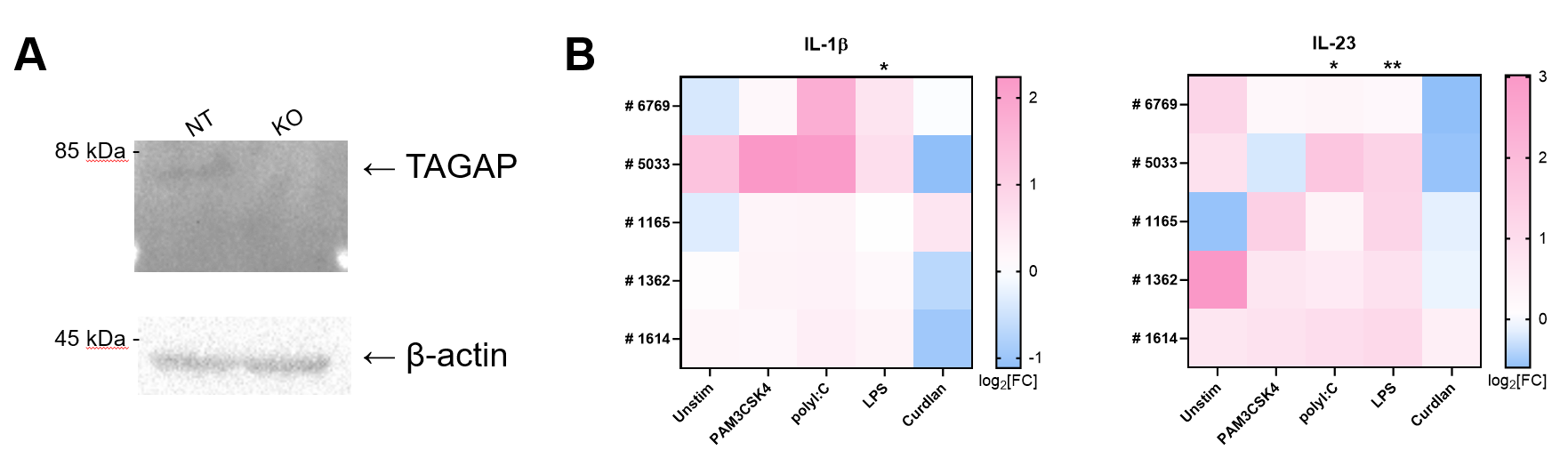
**

**Supplementary Figure 2**

**(A)** Western blotting of TAGAP from human primary monocytes following the CRISPR knockout.

**(B)** Assessment of IL-1β and IL-23 content from human monocyte-derived macrophage cultures. Blood monocytes from two individual donors (#6769, #5033, #1165, #1362, and #1614) were purified, electroporated with non-targeting (NT) or TAGAP sgRNA, and differentiated, followed by 100 ng/ml PAM3CSK4, 100 ng/ml polyI:C, 100 ng/ml LPS, or 100 µg/ml Curdlan stimulation for 24hr. Secretion of the cytokines was quantified using MSD. For each donor and condition, cytokine levels from TAGAP KO cells were normalized to their corresponding NT counterparts. Statistical significance was determined by comparing the KO/NT fold change against a baseline value of 1 (NT/NT). Data are presented as log2 fold changes (KO/NT). (n = 5; **P < 0.01, *P < 0.05 using Student’s t-test).

NT, non-targeting control; KO, TAGAP knockout.

**
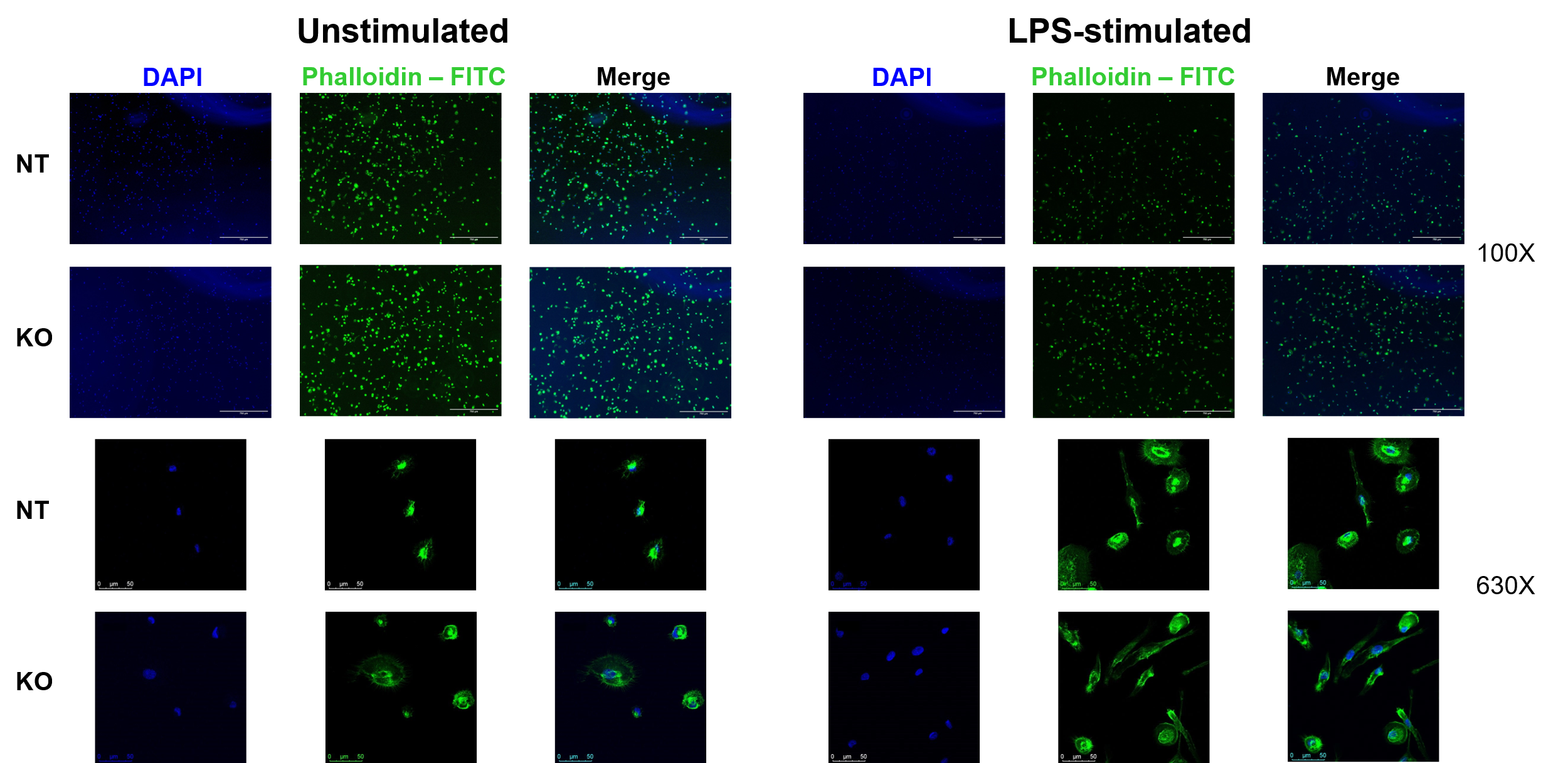
**

**Supplementary Figure 3**

Representative morphologies of non-targeting (control) or TAGAP KO macrophages. Four thousand seeded cells (50% confluency) were analyzed after PBS (unstimulated control) or 100 ng/ml LPS stimulation for 24hr by fluorescent confocal microscopy.

NT, non-targeting control; KO, TAGAP knockout.

**
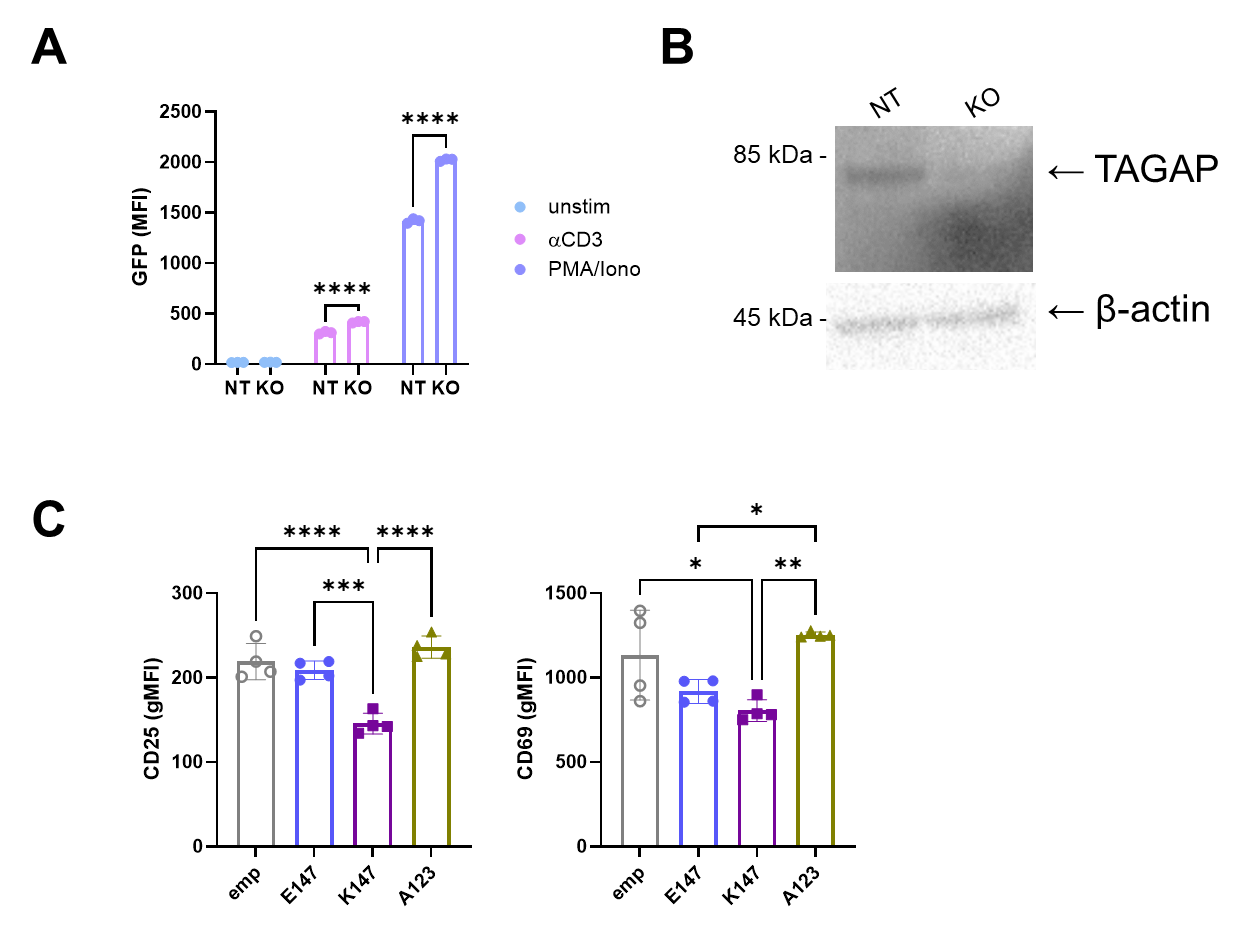
**

**Supplementary Figure 4**

**(A)** The expression of GFP on non-targeting (control) or TAGAP KO Jurkat-NFAT-GFP cells stimulated with 1 µg/ml anti-CD3 antibodies or 10 ng/ml PMA and 500 ng/ml Ionomycin for 24hr (n = 3; ****P < 0.0001 using two-way ANOVA test followed by the post-hoc Fisher’s LSD multiple comparisons test).

**(B)** Western blotting of TAGAP from human primary T cells following the CRISPR knockout.

**(C)** The expression of CD25 and CD69 on TAGAP-deficient Jurkat cells transfected with empty backbone vectors or constructs encoding TAGAP E147, K147, or A123, followed by 1 µg/ml anti-CD3 antibody stimulation for 24hr (n = 4; ****P < 0.0001, ***P < 0.001, **P < 0.01, *P < 0.05 using one-way ANOVA test followed by the post-hoc Sidak multiple comparisons test).

Each dot represents an individual reaction. All data are presented as mean ± SD.

NT, non-targeting control; KO, TAGAP knockout. Iono, ionomycin.


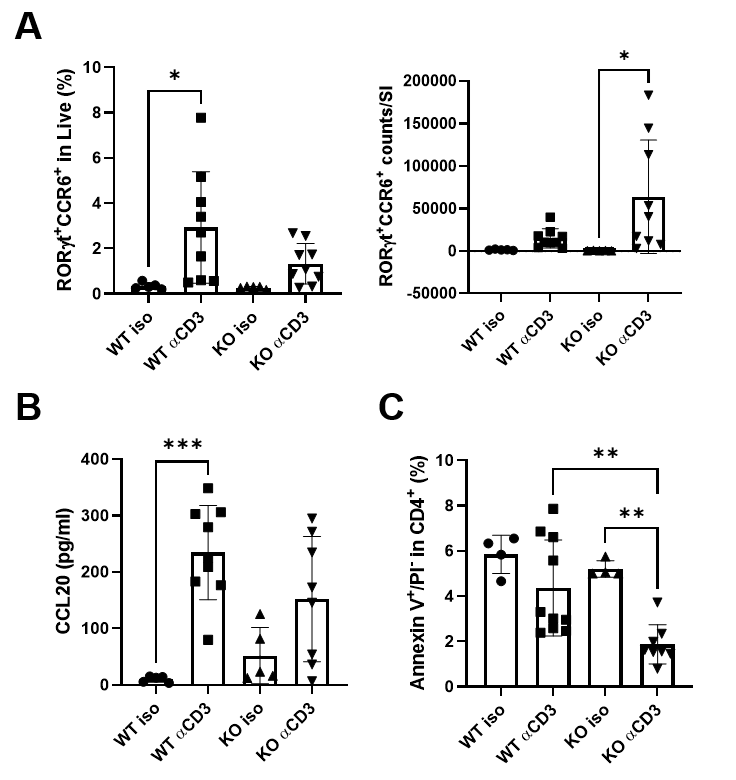


**Supplementary Figure 5**

**(A)** The percentage (left) and counts (right) of RORγt^+^CCR6^+^ cells in SI IEL from WT or TAGAP KO mice subjected to isotype control antibodies or anti-CD3 antibodies (n = 5 or 9; *P < 0.05 using one-way ANOVA test followed by the post-hoc Sidak multiple comparisons test).

**(B)** CCL20 levels in SI tissues from WT or TAGAP KO mice subjected to isotype control antibodies or anti-CD3 antibodies (n = 5 or 9; ***P < 0.001 using one-way ANOVA test followed by the post-hoc Sidak multiple comparisons test).

**(C)** The percentage of apoptotic cell (Annexin V^+^/PI^-^) in SI LP from WT or TAGAP KO mice subjected to isotype control antibodies or anti-CD3 antibodies (n = 5 or 9; **P < 0.01 using one-way ANOVA test followed by the post-hoc Sidak multiple comparisons test).

All n-numbers represent data derived from separate mice with data plotted as mean ± SD.

WT, wild-type; KO, TAGAP-deficient; iso, isotype control antibodies.

**
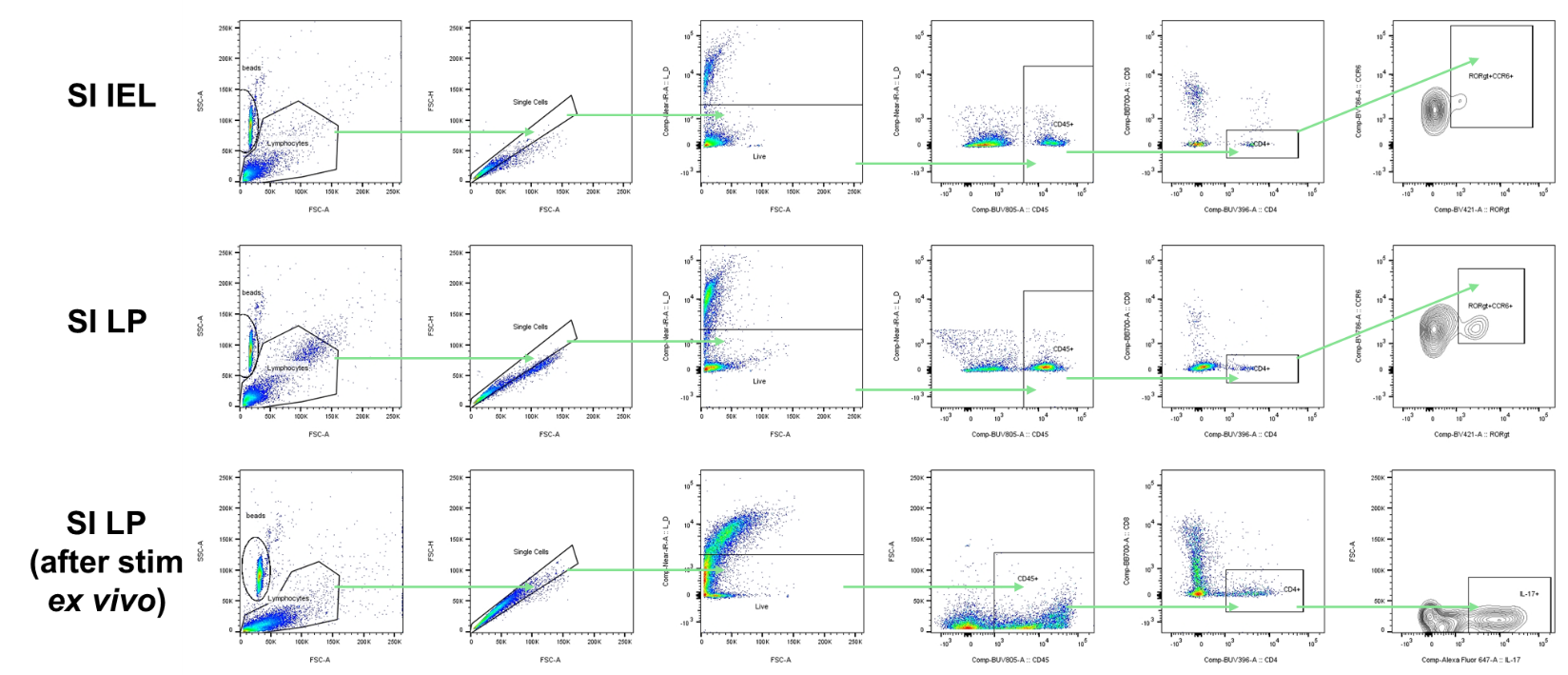
Supplementary Figure 6**

Representative plots illustrating the sequential gating steps used to identify RORγt^+^CCR6^+^ populations in SI IEL and LP and assess IL-17^+^ populations in SI LP post *ex vivo* stimulation from anti-CD3-challenged mice. The gating strategy is adapted from Esplugues *et al.* (Nature, 2012; doi: 10.1038/nature10228). Gates were set based on FMO controls. All plots shown represent samples from a single mouse and are representative of at least three independent experiments.

**Supplementary Figure 7**

The body weight change plot of Rag2^-/-^ mice transfused with WT or TAGAP KO CD4^+^CD45RB^hi^ T cells. Percent change in body weight was monitored every other day for each mouse and normalized to baseline weight measured at Day 0. Data are presented as mean ± SD. (AT WT n = 13 and AT KO n = 11).


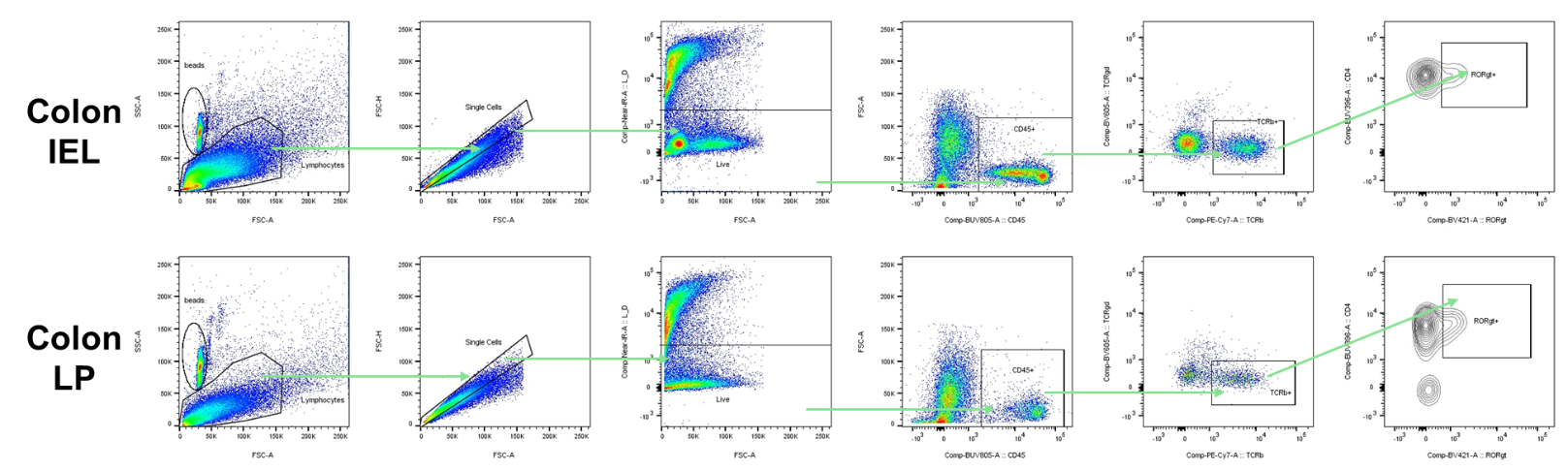
**Supplementary Figure 8**

Representative plots illustrating the sequential gating steps used to identify CD4^+^RORγt^+^ populations in colon IEL and LP from CD4^+^CD45RB^hi^-mediated chronic colitis mice. Gates were set based on FMO controls. All plots shown represent samples from a single mouse and are representative of at least three independent experiments.

**
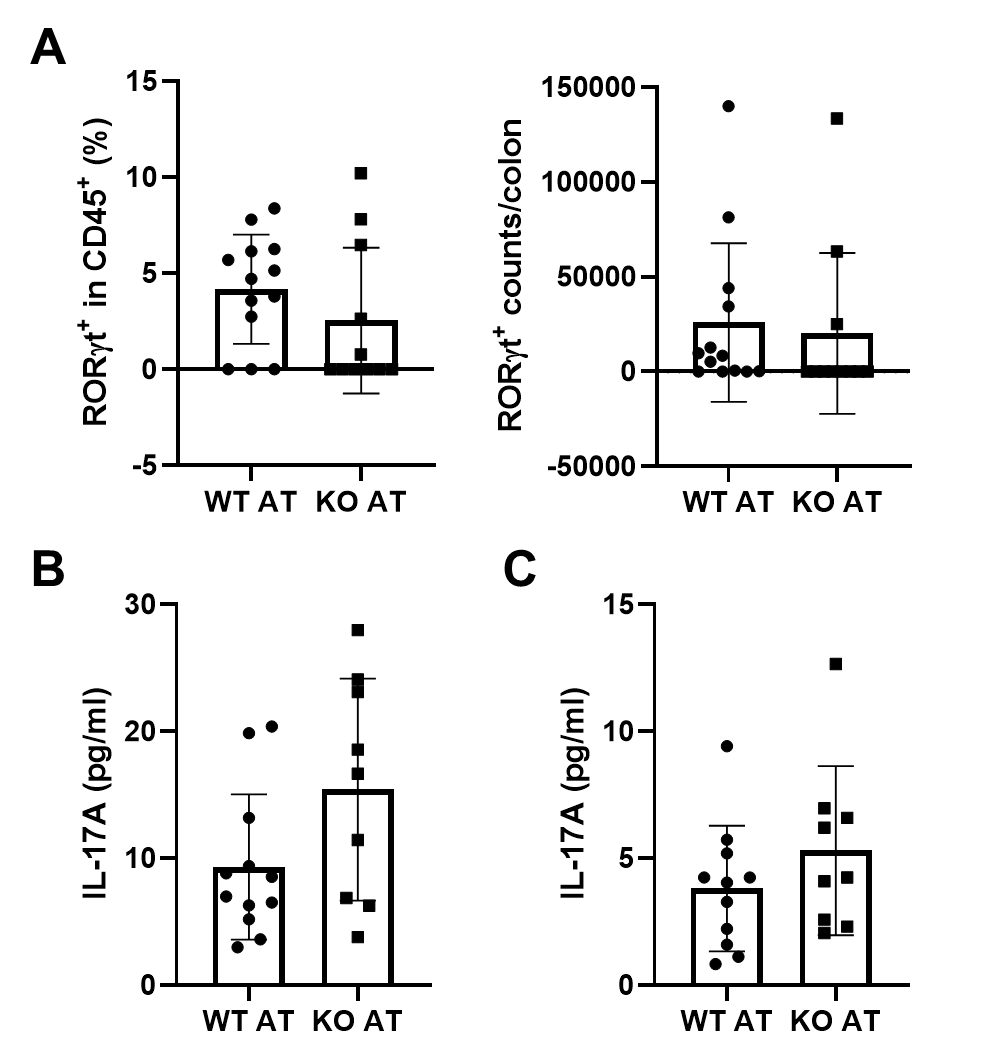
**

**Supplementary Figure 9**

**(A)** The percentage (left) and counts (right) of RORγt^+^ cells in colon LP from Rag2^-/-^ mice transfused with WT or TAGAP KO CD4^+^CD45RB^hi^ T cells (n = 11 or 13).

**(B)** IL-17A levels in serum from Rag2^-/-^ mice transfused with WT or TAGAP KO CD4^+^CD45RB^hi^ T cells (n = 11 or 13; P value was calculated using Student’s t-test).

**(C)** IL-17A levels in colon tissue from Rag2^-/-^ mice transfused with WT or TAGAP KO CD4^+^CD45RB^hi^ T cells (n = 11 or 13; P value was calculated using Student’s t-test).

All n-numbers represent data derived from separate mice with data plotted as mean ± SD.

WT AT, adoptively transferred with wild-type CD4^+^CD45RB^hi^ T cells; KO AT, adoptively transferred with TAGAP-deficient CD4^+^CD45RB^hi^ T cells.

**Supplementary Table 1. Sequence of human recombinant TAGAP proteins**

| **Name** | **Sequence** |
| --- | --- |
| **WT**  **E147** | MHHHHHHGSGKIEEGKLVIWINGDKGYNGLAEVGKKFEKDTGIKVTVEHPDKLEEKFPQVAATGDGPDIIFWAHDRFGGYAQSGLLAEITPDKAFQDKLYPFTWDAVRYNGKLIAYPIAVEALSLIYNKDLLPNPPKTWEEIPALDKELKAKGKSALMFNLQEPYFTWPLIAADGGYAFKYENGKYDIKDVGVDNAGAKAGLTFLVDLIKNKHMNADTDYSIAEAAFNKGETAMTINGPWAWSNIDTSKVNYGVTVLPTFKGQPSKPFVGVLSAGINAASPNKELAKEFLENYLLTDEGLEAVNKDKPLGAVALKSYEEELAKDPRIAATMENAQKGEIMPNIPQMSAFWYAVRTAVINAASGRQTVDEALKDAQTNSSSNNNNNNNNNNENLYFQGMKLRSSHNASKTLNANNMETLIECQSEGDIKEHPLLASCESEDSICQLIEVKKRKKVLSWPFLMRRLSPASDFSGALETDLKASLFDQPLSIICGDSDTLPRPIQDILTILCLKGPSTEGIFRRAANEKARKELKEELNSGDAVDLERLPVHLLAVVFKDFLRSIPRKLLSSDLFEEWMGALEMQDEEDRIEALKQVADKLPRPNLLLLKHLVYVLHLISKNSEVNRMDSSNLAICIGPNMLTLENDQSLSFEAQKDLNNKVKTLVEFLIDNCFEIFGENIPVHSSITSDDSLEHTDSSDVSTLQNDSAYDSNDPDVESNSSSGISSPSRQPQVPMATAAGLDSAGPQDAREVSPEPIVSTVARLKSSLAQPDRRYSEPSMPSSQECLESRVTNQTLTKSEGDFPVPRVGSRLESEEAEDPFPEEVFPAVQGKTKRPVDLKIKNLAPGSVLPRALVLKAFSSSSLDASSDSSPVASPSSPKRNFFSRHQSFTTKTEKGKPSREIKKHSMSFTFAPHKKVLTKNLSAGSGKSQDFTRDHVPRGVRKESQLAGRIVQENGCETHNQTARGFCLRPHALSVDDVFQGADWERPGSPPSYEEAMQGPAARLVASESQTVGSMTVGSMRARMLEAHCLLPPLPPAHHVEDSRHRGSKEPLPGHGLSPLPERWKQSRTVHASGDSLGHVSGPGRPELLPLRTVSESVQRNKRDCLVRRCSQPVFEADQFQYAKESYIGSDYKDDDDK* |
| **K147** | MHHHHHHGSGKIEEGKLVIWINGDKGYNGLAEVGKKFEKDTGIKVTVEHPDKLEEKFPQVAATGDGPDIIFWAHDRFGGYAQSGLLAEITPDKAFQDKLYPFTWDAVRYNGKLIAYPIAVEALSLIYNKDLLPNPPKTWEEIPALDKELKAKGKSALMFNLQEPYFTWPLIAADGGYAFKYENGKYDIKDVGVDNAGAKAGLTFLVDLIKNKHMNADTDYSIAEAAFNKGETAMTINGPWAWSNIDTSKVNYGVTVLPTFKGQPSKPFVGVLSAGINAASPNKELAKEFLENYLLTDEGLEAVNKDKPLGAVALKSYEEELAKDPRIAATMENAQKGEIMPNIPQMSAFWYAVRTAVINAASGRQTVDEALKDAQTNSSSNNNNNNNNNNENLYFQGMKLRSSHNASKTLNANNMETLIECQSEGDIKEHPLLASCESEDSICQLIEVKKRKKVLSWPFLMRRLSPASDFSGALETDLKASLFDQPLSIICGDSDTLPRPIQDILTILCLKGPSTEGIFRRAANEKARKELKEELNSGDAVDLKRLPVHLLAVVFKDFLRSIPRKLLSSDLFEEWMGALEMQDEEDRIEALKQVADKLPRPNLLLLKHLVYVLHLISKNSEVNRMDSSNLAICIGPNMLTLENDQSLSFEAQKDLNNKVKTLVEFLIDNCFEIFGENIPVHSSITSDDSLEHTDSSDVSTLQNDSAYDSNDPDVESNSSSGISSPSRQPQVPMATAAGLDSAGPQDAREVSPEPIVSTVARLKSSLAQPDRRYSEPSMPSSQECLESRVTNQTLTKSEGDFPVPRVGSRLESEEAEDPFPEEVFPAVQGKTKRPVDLKIKNLAPGSVLPRALVLKAFSSSSLDASSDSSPVASPSSPKRNFFSRHQSFTTKTEKGKPSREIKKHSMSFTFAPHKKVLTKNLSAGSGKSQDFTRDHVPRGVRKESQLAGRIVQENGCETHNQTARGFCLRPHALSVDDVFQGADWERPGSPPSYEEAMQGPAARLVASESQTVGSMTVGSMRARMLEAHCLLPPLPPAHHVEDSRHRGSKEPLPGHGLSPLPERWKQSRTVHASGDSLGHVSGPGRPELLPLRTVSESVQRNKRDCLVRRCSQPVFEADQFQYAKESYIGSDYKDDDDK* |
| **A123** | MHHHHHHGSGKIEEGKLVIWINGDKGYNGLAEVGKKFEKDTGIKVTVEHPDKLEEKFPQVAATGDGPDIIFWAHDRFGGYAQSGLLAEITPDKAFQDKLYPFTWDAVRYNGKLIAYPIAVEALSLIYNKDLLPNPPKTWEEIPALDKELKAKGKSALMFNLQEPYFTWPLIAADGGYAFKYENGKYDIKDVGVDNAGAKAGLTFLVDLIKNKHMNADTDYSIAEAAFNKGETAMTINGPWAWSNIDTSKVNYGVTVLPTFKGQPSKPFVGVLSAGINAASPNKELAKEFLENYLLTDEGLEAVNKDKPLGAVALKSYEEELAKDPRIAATMENAQKGEIMPNIPQMSAFWYAVRTAVINAASGRQTVDEALKDAQTNSSSNNNNNNNNNNENLYFQGMKLRSSHNASKTLNANNMETLIECQSEGDIKEHPLLASCESEDSICQLIEVKKRKKVLSWPFLMRRLSPASDFSGALETDLKASLFDQPLSIICGDSDTLPRPIQDILTILCLKGPSTEGIFARAANEKARKELKEELNSGDAVDLERLPVHLLAVVFKDFLRSIPRKLLSSDLFEEWMGALEMQDEEDRIEALKQVADKLPRPNLLLLKHLVYVLHLISKNSEVNRMDSSNLAICIGPNMLTLENDQSLSFEAQKDLNNKVKTLVEFLIDNCFEIFGENIPVHSSITSDDSLEHTDSSDVSTLQNDSAYDSNDPDVESNSSSGISSPSRQPQVPMATAAGLDSAGPQDAREVSPEPIVSTVARLKSSLAQPDRRYSEPSMPSSQECLESRVTNQTLTKSEGDFPVPRVGSRLESEEAEDPFPEEVFPAVQGKTKRPVDLKIKNLAPGSVLPRALVLKAFSSSSLDASSDSSPVASPSSPKRNFFSRHQSFTTKTEKGKPSREIKKHSMSFTFAPHKKVLTKNLSAGSGKSQDFTRDHVPRGVRKESQLAGRIVQENGCETHNQTARGFCLRPHALSVDDVFQGADWERPGSPPSYEEAMQGPAARLVASESQTVGSMTVGSMRARMLEAHCLLPPLPPAHHVEDSRHRGSKEPLPGHGLSPLPERWKQSRTVHASGDSLGHVSGPGRPELLPLRTVSESVQRNKRDCLVRRCSQPVFEADQFQYAKESYIGSDYKDDDDK* |

**Supplementary Table 2. List of antibodies**

| **Product** | **Catalog #** | **Vendor** | **Dilution** |
| --- | --- | --- | --- |
| **Anti-TAGAP antibody** | ab187664 | abcam | 1:1000 |
| **Anti-TAGAP antibody** | NBP3-22603 | NOVUS | 1:1000 |
| **Anti-beta actin Antibody-HRP** | MA515739HRP | Invitrogen | 1:10000 |
| **BB515 anti-human CD25** | 564467 | BD Biosciences | 1:100 |
| **PE/Cyanine7 anti-human CD69** | 310912 | Biolegend | 1:100 |
| **BUV805 anti-mouse CD45** | 748370 | BD Biosciences | 1:100 |
| **BUV395 anti-mouse CD4** | 568375 | BD Biosciences | 1:100 |
| **Alexa Fluor 647 anti-mouse IL-17A antibody** | 506912 | Biolegend | 1:100 |
| **BV421 mouse anti-mouse RORγt** | 562894 | BD Biosciences | 1:100 |
| **Brilliant Violet 785 anti-mouse CD196 (CCR6) antibody** | 129823 | Biolegend | 1:100 |

**Supplementary Table 3. Sequence of non-targeting (NT) and TAGAP sgRNA**

| **Name** | **Sequence** |
| --- | --- |
| **NT sgRNA-1** | GTGGACGGTCGTGCGCTGTG |
| **NT sgRNA-2** | GCACUACCAGAGCUAACUCA |
| **TAGAP sgRNA-1** | CTTAAAGGCCCTTCAACGGA |
| **TAGAP sgRNA-2** | ACTTTCAAGCGACCTCTTTG |
| **TAGAP sgRNA-3** | AGACCAAGTGCTTGAGTAGC |

**Supplementary Table 4. List of TLR and Dectin-1 ligands**

| **Product** | **Catalog #** | **Vendor** |
| --- | --- | --- |
| **Pam3CSK4** | tlrl-pms | InvivoGen |
| **Poly(I:C)** | tlrl-pic | InvivoGen |
| **LPS** | tlrl-eblps | InvivoGen |
| **Curdlan** | tlrl-curd | InvivoGen |
